# Aerogel-Based Materials in Bone and Cartilage Tissue Engineering—A Review with Future Implications

**DOI:** 10.3390/gels9090746

**Published:** 2023-09-13

**Authors:** István Lázár, Ladislav Čelko, Melita Menelaou

**Affiliations:** 1 Department of Inorganic and Analytical Chemistry, University of Debrecen, Egyetem tér 1, 4032 Debrecen, Hungary; 2 Central European Institute of Technology, Brno University of Technology, Purkynova 656/123, 612 00 Brno, Czech Republic; ladislav.celko@ceitec.vutbr.cz; 3 Department of Chemical Engineering, Cyprus University of Technology, 30 Arch. Kyprianos Str., Limassol 3036, Cyprus

**Keywords:** aerogel, tissue engineering, artificial bone substitution, in vitro and in vivo bioactivity, biodegradation, cartilage regeneration, scaffold, osteogenesis, simulated body fluids, immortalized cell lines

## Abstract

Aerogels are fascinating solid materials known for their highly porous nanostructure and exceptional physical, chemical, and mechanical properties. They show great promise in various technological and biomedical applications, including tissue engineering, and bone and cartilage substitution. To evaluate the bioactivity of bone substitutes, researchers typically conduct in vitro tests using simulated body fluids and specific cell lines, while in vivo testing involves the study of materials in different animal species. In this context, our primary focus is to investigate the applications of different types of aerogels, considering their specific materials, microstructure, and porosity in the field of bone and cartilage tissue engineering. From clinically approved materials to experimental aerogels, we present a comprehensive list and summary of various aerogel building blocks and their biological activities. Additionally, we explore how the complexity of aerogel scaffolds influences their in vivo performance, ranging from simple single-component or hybrid aerogels to more intricate and organized structures. We also discuss commonly used formulation and drying methods in aerogel chemistry, including molding, freeze casting, supercritical foaming, freeze drying, subcritical, and supercritical drying techniques. These techniques play a crucial role in shaping aerogels for specific applications. Alongside the progress made, we acknowledge the challenges ahead and assess the near and far future of aerogel-based hard tissue engineering materials, as well as their potential connection with emerging healing techniques.

## 1. Introduction

Bone is a rigid tissue with essential functions in providing structural support, protecting vital organs, and enabling movement [1]. It consists of an organic matrix (20%), primarily made up of type I collagen, a mineral phase (65%) predominantly composed of hydroxyapatite [Ca_10_(PO_4_)_6_(OH)_2_, HAp], water (10%), and various bioactive factors and cells, mainly osteoblasts and osteoclasts [2]. Bone has a natural regenerative process that is regulated by biomechanical, cellular, and molecular factors [3]. Articular cartilage is a thin layer covering the ends of bones, allowing smooth gliding and facilitating proper joint function. The cartilage tissue is a sturdy, flexible avascular structure composed of collagen, proteoglycan, non-collagenous proteins, and water. A unique feature of cartilage is its close connection with the underlying hard subchondral bone, comprising three distinct components: highly mineralized subchondral, intermediate-mineralized calcified, and non-mineralized tissues, separated by a dense tidemark.

Damage to bones and cartilage often occurs due to disease or traumatic injuries. Incidences of bone- and cartilage-related disorders have been on the rise, linked to factors like aging, obesity, cancer, and sports-related injuries. These conditions can significantly impact patients’ quality of life, causing pain, reduced mobility, and loss of independence. To address these challenges, the research community has been focusing on regenerative medicine approaches, including the development of biomaterial-based and tissue-engineering solutions.

Restoring bone integrity and structure is essential in cases of fractures, skeletal development, or regular physiological reshaping. It involves facilitating the transport, growth, proliferation, and differentiation of osteoprogenitor cells in the injured or defective area. To achieve successful bone repair, a well-designed system is necessary to support the three primary mechanisms of bone regeneration: (a) rapid revascularization, (b) osteogenesis induction, and (c) osteoinduction, which generate new tissue from osteogenic cells. Additionally, the process should promote osteoconduction and encourage cell growth towards the bone surface [4]. However, there are instances where bone regeneration or repair exceeds the tissue’s capacity for new bone formation. Such cases may include bone deformations, neoplastic diseases, infections, avascular necrosis, and osteoporosis, among others [3].

Addressing bone defects requires orthopedic reconstruction methodologies that involve bone replacement through the implantation of natural or artificial grafts [5]. Both types of grafts must meet specific criteria, such as high biocompatibility, osteoconduction, and osseointegration [6]. Moreover, they should exhibit robust mechanical strength, be harmless to the body, and remain stable in the biological environment. Throughout their presence in the body, the grafts must not demonstrate any toxic effects [5].

Recent advancements in bone tissue engineering are centered around the development of structures that can closely mimic the behavior of natural bone in terms of both structure and performance. This involves creating materials with exceptional mechanical reinforcement and a supporting matrix, all while maintaining biocompatibility [7]. To promote successful regeneration, these materials need to be highly porous, encouraging vascularization into the damaged area and facilitating the migration of osteogenic cells. Biocompatible three-dimensional scaffolds or hydrogels often possess these desirable characteristics [8].

Scaffolds tailored for bone and cartilage tissue engineering must meet specific biological requirements. They should be biocompatible, non-toxic, and biodegradable, capable of seamlessly integrating and interacting with the surrounding environment. Porosity is a key factor, as it enables cellular infiltration and facilitates the transport of essential gases, nutrients, and regulatory factors, all crucial for cell survival. Finding the right balance is crucial, as excessively large pores reduce the surface area available for cell attachment, while overly small pores hinder cell migration and infiltration, and restrict the diffusion of vital nutrients and waste products. Research conducted by Matsiko et al. suggests that the optimal pore sizes for bone and cartilage tissue-engineering applications typically fall within the range of 100–300 μm [9]. Various fabrication methods can be employed to create these scaffolds, such as freeze drying, electrospinning, and 3D printing, as studied by Iglesias-Mejuto, García-González, Włodarczyk-Biegun, and del Campo [10,11]. Detailed insights into these methods will be provided in Section 3.

In bone and cartilage repair applications, both synthetic and natural polymers have found utility. Synthetic polymers offer versatility in their physical and chemical properties, allowing precise control over molecular weight, degradation time, and hydrophobicity. Prominent examples of synthetic materials employed in such applications, as reported by Puppi et al., include poly L-lactic acid (PLLA), polycaprolactone (PCL), polyglycolic acid (PGA), and polyethylene glycol (PEG) [12]. These synthetic materials can be used in various forms to create scaffolds of different shapes and sizes.

Conversely, natural polymers boast several advantages over their synthetic counterparts. They demonstrate biocompatibility, biodegradability with non-toxic degradation products, and possess bioactive properties that facilitate enhanced cell interactions. Some of the natural polymers used for bone and cartilage repair applications include collagen, silk, gelatin, fibrinogen, elastin, keratin, actin, and myosin. Several examples of polysaccharide-based aerogels exist, such as the crosslinked cellulose nanocrystal aerogels synthesized by Osorio et al. [13], an alginate aerogel reported by Wu et al. [14], and the development of a novel high-methoxyl pectin–xanthan aerogel coating on medical-grade stainless steel reported by Horvat et al. [15]. They can be classified as “bio-aerogels”, which originated from natural, semi-synthetic, and synthetic sources, with promising biomedical applications. The processing steps of the polysaccharide-based aerogels are similar to those applied for silica and other organic counterparts and, most commonly, start with the preparation of gel from an aqueous solution (often called hydrogel or “aquagel”) and the water in the pores of the aquagel is replaced with an alcohol such as methanol, to prepare an “alcogel” and to make possible the drying by supercritical carbon dioxide (scCO_2_) [16]. Alternatively, the solvent exchange step can be circumvented if gelation is directly carried out in alcohol. In addition, composite materials such as PEGDA/CNF aerogel–wet hydrogel scaffold (where PEGDA: polyethylene glycol diacrylate; and CNF: cellulose nanofibril) have been proposed to overcome limitations of the single components in a concise review by Kazimierczak and Przekora [4].

Ceramic materials in the form of calcium phosphate have also been studied and proposed as potential candidates for bone regeneration and/or substitution. Hydroxyapatite (HAp), the main inorganic constituent of human bone, has high biocompatibility and osteo-conductivity, rendering it a material of particular interest for bone regeneration [3]. HAp biomaterials, however, are characterized by poor cell adhesion and difficult ingrowth, thus limiting their therapeutic effect in clinical applications. Also, it is not easy to prepare single-phase HAp porous scaffolds with both high porosity and excellent mechanical properties to be suitable candidates for bone regeneration. Thus, researchers worldwide are working to improve the properties of such materials. An example includes the work of Duan and coworkers who investigated HAp-based composite porous scaffolds instead of “HAp-only” porous scaffolds for such applications. Their experimental results suggest that they prepared a promising material in the form of HAp nanowire aerogel scaffold [17].

Other promising calcium phosphate-based ceramic materials are the a-tricalcium phosphate (α-Ca_3_(PO_4_)_2_, α-TCP) and β-tricalcium phosphate (β-Ca_3_(PO_4_)_2_, β-TCP). Combining the excellent biocompatibility of β-TCP and the conductivity of carbon aerogels, Tevlek et al. synthesized a β-TCP carbon–aerogel composite material. The biocompatibility of the composite material was evaluated, and their results suggested that composites may also act as promising targets for such applications [18]. Also, Lin and coworkers developed a β-TCP bioceramic platform coated with carbon aerogel as a novel approach to conquer osteosarcoma in one step [19].

In 2015, Wan et al. proposed mesoporous TiO_2_ nanotube materials as a novel 3D porous network-structured scaffold for potential bone tissue engineering. The TiO_2_ nanotubes were synthesized using the template-assisted sol–gel method followed by calcination. The scaffold showed an extremely large surface area of 1629 m^2^ g^−1^ and a diameter of less than 100 nm [20].

Repairing cartilage defects remains a significant challenge in the field. While various clinical treatments for cartilage regeneration, such as microfracture, autologous chondrocyte implantation, Pridie perforations, and transplantation of osteochondral plugs have been developed [21], their success in fully regenerating functional cartilage tissue has been limited. To address these limitations, alternative approaches have been proposed, including the use of cell-loaded scaffold constructs. For successful cartilage regeneration through tissue engineering, an ideal scaffold must possess certain crucial characteristics, similar to those required for bone regeneration. These characteristics include a biomimetic three-dimensional (3D) architecture to facilitate cell adhesion, an appropriate porosity to support cell ingrowth, sufficient mechanical strength to maintain its shape, good biocompatibility, and biodegradability, among others. These essential features are key to developing effective strategies for cartilage repair and regeneration.

Electrospinning proves to be a highly effective technique for producing composite fibers with varying diameters and arrangements, closely resembling the morphology of the natural extracellular matrix (ECM) found in cartilage tissue, while also possessing suitable mechanical properties. For instance, Feng et al. explored a novel method involving electrospinning cartilage-derived extracellular matrix and polycaprolactone (PCL) composite nanofibrous membranes [22]. The traditional electrospinning technique primarily produces two-dimensional (2D) fiber membrane materials with minimal thickness and small pores. However, researchers have pursued the electrospinning of multi-component nanofibers to overcome the limitations associated with individual polymers and to cater to specific requirements. These requirements encompass crucial aspects like mechanical strength, biocompatibility, and degradation rate. By finely adjusting the proportion of each component in the composite fibers, these special requirements can be met, as was reviewed in detail by Chen et al. [23].

Various methods for preparing 3D electrospun nanofibrous scaffolds have been extensively researched and published. Examples include multilayering electrospinning, as reported by Zhang et al. [24] and Chainani et al. [25], as well as liquid and template-assisted electrospinning and post-treated electrospinning, as explored by Shim and colleagues [26], among others. In light of these advancements, Chen et al. [23] and Li et al. [27] successfully prepared aerogels composed of electrospun gelatin/polylactide (Gel/PLA) or gelatin/polycaprolactone (Gel/PCL) fibers, offering promising potential for cartilage regeneration. Additionally, Wang et al. developed a 3D fibrous aerogel comprising SiO_2_ nanofibers with chitosan serving as bonding sites for bone regeneration [28]. These reports represent a few examples demonstrating the feasibility and potential of fibrous aerogels in the fields of cartilage and bone tissue engineering. Furthermore, inorganic components like hydroxyapatite (HAp) have been widely incorporated into implants for calcified cartilage and subchondral bone regeneration. Meanwhile, glycosaminoglycans (GAG) such as hyaluronic acid (HA) and chondroitin sulfate (CS) are frequently utilized for cartilage regeneration. These materials play crucial roles in enhancing the performance and functionality of tissue-engineering scaffolds.

The quest for robust and long-lasting bone regeneration and cartilage tissue remains an important and challenging topic. In light of this, the present review article offers an overview of (composite) aerogel materials, a remarkable category of nanoporous materials with great potential for bone and cartilage repair applications. In the following sections, detailed information on the physical and chemical properties, and the significant role that such aerogels can play in various bone-related biomedical applications will be explored.

## 2. Aerogel Microstructure

The discovery of aerogels dates back to 1931 when Kistler published the first article on the subject in Nature, titled “Coherent expanded aerogels and jellies” [29]. Kistler’s groundbreaking work involved the successful synthesis of aerogels from silica, achieved through the condensation of sodium metasilicate. He later expanded his research to include aerogels made from alumina, tungsten, nickel tartrate, cellulose, and gelatin. In his definition, aerogels were described as “gels in which the liquid has been replaced by air, with moderate shrinkage of the solid network.” For over 50 years, aerogels received little attention from the scientific community. However, in the last four decades, the interest in these materials has grown exponentially. This surge in interest can be attributed to the diverse range of applications that aerogels offer in various fields. Notably, aerogels provided solutions in catalysis, aerospace, and construction industries, for example. They have also proven valuable in energy-storage devices, solar-steam generation, and medical applications.

Aerogels are remarkable porous ultralight solid materials obtained from gels, wherein the liquid component is replaced by a gas, commonly air. These aerogels exhibit several distinctive characteristics, including (a) high porosity (exceeding 90% of the total volume), (b) very low apparent density, (c) very high specific surface area, and (d) high mechanical strength when compared to the density of the material.

The term “aerogel” encompasses a broad description of the structure and does not impose specific restrictions on material compositions or synthetic methodologies. Hüsing and Schubert proposed that “aerogels are materials in which the typical pore structures and networks remain remarkably maintained when the pore liquid of a gel is replaced by air” [30]. This definition better captures the essential characteristic of aerogels, highlighting their porous and highly structured nature, even after the liquid component has been replaced by air.

In this regard, aerogel materials exhibit a wide range of classifications, as discussed by Karamikamkar et al. [31]. These classifications include their appearance, microstructural characteristics, composition, polarity and surface functionality.

The most commonly employed and, perhaps, the simplest method for producing aerogels is the well-established sol–gel approach, followed by the specific drying process, known as supercritical drying at or above the supercritical point [30]. The sol–gel procedure involves two main stages: the formation of a sol and the subsequent transformation into a gel. The last step involves removing the pore liquid through a specialized drying process, leading to the formation of the aerogel. This drying step plays a critical role in shaping the final physical and chemical profile of the aerogel, allowing for precise control over its characteristics and performance.

**Figure 1:**
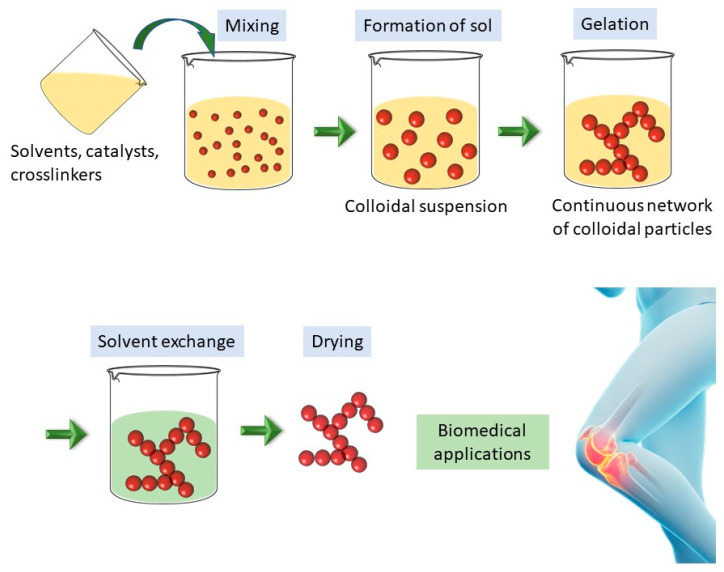
Graphical visualization of the general process of making aerogels in a sol–gel process followed by a specific drying technique to provide aerogel materials for biomedical applications.

The gelation of inorganic aerogels primarily relies on hydrolysis and condensation processes, while biopolymer aerogels form through the aggregation process. Subsequent to gel formation, liquid extraction from the gel can be achieved using various techniques, resulting in materials classified as xerogels, cryogels, and aerogels [32]. The drying methods strongly affect the final properties of these materials, and are discussed in Section 3. (Figure 1)

To address drawbacks like mechanical issues and limited specific functionalities, various new synthetic approaches have been employed in the production of aerogels. Techniques such as ambient pressure drying and freeze drying have been utilized to tailor the physical, chemical, and biological properties of aerogels, leading to the design and synthesis of hybrid inorganic or organic–inorganic hybrid aerogels. Aerogels stand apart from conventional foams due to their nanometer-scale pores with intricate interconnectivity, resulting in their superior insulating capabilities, being 2–5 times more effective than foams, with low thermal conductivity (0.005–0.1 W/mK), and an ultra-low dielectric constant (k = 1.0–2.0). Such characteristics make aerogels highly appealing for various applications. However, one of the challenges faced by these materials lies in their mechanical properties, which can be limited. For instance, silica aerogels are known for their fragility, hygroscopic nature, and poor mechanical properties, leading to drawbacks in certain applications [31]. To expand the range of applications while preserving the unique properties of aerogels, mechanical reinforcing strategies have been devised. For silica-based aerogels, which represent the most extensively studied family, several methods have been explored in the literature to improve their mechanical properties. A common technique employed for structural reinforcement is prolonged aging time, as utilized by Hong et al., leading to the development of 3D internetworked GA@PDMS (where GA: graphene aerogel; and PDMS: poly(dimethylsiloxane)) [33]. Another widely employed approach involves surface-crosslinking of a silica backbone with a polymer. Boday et al. demonstrated the growth of silica aerogel polymer nanocomposites in the presence of poly(methyl methacrylate) (PMMA), while Leventis reported the development of silica aerogels crosslinked with isocyanate-derived polymers [34,35]. In addition, the incorporation of a secondary phase, such as an organic/inorganic phase, embedded in the structure before (or after) gelation, has proven to be an effective strategy for aerogel structural reinforcement, as demonstrated by Randall and coworkers [36]. Theoretical considerations also suggest that improving elastic recovery in silica aerogels can be achieved by including organic flexible linking groups in the silica backbone or by crosslinking the underlying structural gel with silanol groups through reactions with precursors, monomers, or polymers, as described by Lenentis et al. [37]. These methodologies have successfully enhanced the mechanical properties of aerogels while also improving their transparency.

## 3. Formulation and Drying Methods

### 3.1. Formulation Methods

#### 3.1.1. Casting, Molding

Monolithic aerogels are crafted through a straightforward casting or molding technique, which stands as the most extensively employed procedure. The constituents for gel formation are poured in a suitable container, allowing the gelation process to reach completion while facilitating subsequent retrieval of the gelled or solidified material devoid of structural compromise. Following this, the material undergoes a series of solvent exchange steps, wherein the original solvent mixture is replaced with an organic solvent compatible with carbon dioxide, such as acetone, methanol, or ethanol. As an alternative route, water-based gels are subjected to freezing and subsequent freeze drying. A viable realization of the casting, solvent exchange, and supercritical drying sequence is elucidated within the literature [38].

#### 3.1.2. Freeze Casting

The freeze-casting process is also frequently used in fabricating porous materials, including aerogels. The technique is thoroughly described in the literature by Li et al. and García-González et al. [39,40]. The aqueous gel is frozen slowly in a segmented pattern before drying. In the process, ice crystals of different sizes are formed in the segmented temperature zones, leading to a patterned meso/macro porosity of the aerogel (cryogel) monoliths after drying, as presented by Tetik and coworkers [41]. This method was used to prepare silk fibroin–silica aerogels by Maleki and coworkers [42] and crosslinked cellulose [13] aerogels for bone substitution by Osorio et al.

#### 3.1.3. Supercritical Foaming

In some instances, supercritical carbon dioxide can also generate gelation and macropore formation. The generally used supercritical foaming technique is reviewed in the literature [43,44] and has been successfully applied for the preparation of aerogel–polymer composite scaffolds made from starch and polycaprolactone by Goimil et al. [45] or from silk fibroin/polycaprolactone by Goimil and coworkers [46].

#### 3.1.4. Stereolithography, 3D Printing

Bio-ink technology and 3D printing stand as firmly established and widely embraced methodologies in the biomedical sphere, particularly in scaffold formulation and the provision of intricate structural arrangements. The outcome of a specific tissue replacement or tissue-mimicking application is contingent upon the materials’ intrinsic nature, the 3D architecture of the scaffold, the involved cell types, and the presence or absence of stimulating factors. Computer-aided design tools rapidly generate the blueprint for a 3D framework, subsequently realized using specialized extrusion or syringe-type printers. These printers can utilize a singular bio-ink component capable of light-induced crosslinking, a pliable yet self-supporting paste that undergoes post-printing crosslinking, or a printer with a two-component coaxial head that triggers chemical reactions upon contact, or even a blend thereof. The fabrication of scaffolds for artificial bone or cartilage substitutes presents challenges due to the intricacies of identifying a suitable 3D-printable material. In biomedical practice, nanofibrous bioactive substances are frequently 3D printed and subsequently subjected to freeze drying, transforming them into aerogels or aerogel-like forms to maintain their structural integrity and functionality. An evaluation of the technique’s merits and limitations has been comprehensively compiled by Badhe and colleagues [47].

Iglesias-Mejuto and García-González prepared an alginate–hydroxyapatite 3D-printed aerogel scaffold [10] as well as sterile dual crosslinked alginate–hydroxyapatite 3D-printed aerogel scaffolds with carbon dioxide gelling and glutaraldehyde crosslinking technology for bone tissue engineering. The as-prepared scaffolds showed enhanced fibroblast migration and good bioactivity; the latter correlated with the hydroxyapatite content [48].

Ng and coworkers developed a technique in which simultaneous 365 nm photo-crosslinking and microextrusion 3D printing of the mixture of methacrylated silk fibroin and methacrylated hollow silica nanoparticles provided a mechanically stable scaffold compared to the simple silk fibroin networks. Unidirectional freeze casting provided even more interconnection of the pores, after which the aerogel was made by freeze drying. The as-prepared material is expected to be osteoconductive and osteoinductive bone substitute material that can be loaded with ciprofloxacin or other drugs to treat bone-related diseases [49].

### 3.2. Drying Methods

Regardless of the specific synthetic methods employed, wet gels undergo diverse drying techniques to transform into aerogel-based materials. Among these approaches, freeze drying and supercritical carbon dioxide drying emerge as the most prevalent. To a somewhat lesser extent, alternative strategies such as subcritical drying, spring-back drying [50], and ambient pressure drying [51] have also been explored and subjected to systematic investigation.

#### 3.2.1. Freeze Drying

Aqueous gels have been effectively transformed into aerogels through freeze drying, often referred to as cryogels. This method can be directly applied to aqueous gels, eliminating the need for the solvent exchange steps necessary in supercritical drying. An inherent benefit is that even highly heat-sensitive materials can be dried without undergoing decomposition. Thus, the freeze-drying technique has been harnessed to craft aerogels with successful outcomes.

Examples include the development of aerogels from nanocellulose-PEGDA by Tang et al. [52], from rGO-collagen by Bahrami et al. [53], from rGO network by Asha et al. [54], from PEGDA-CNF by Sun et al. [55], from nanocellulose–bioglass by Ferreira et al. [56], from CA and PCL nanofiber-reinforced chitosan by Zhang et al. [57], from crosslinked cellulose by Osorio et al. [13], and from silk fibroin–cellulose developed by Chen and coworkers [58]. In a number of cases, freeze drying was combined with a freeze-casting/cryotemplating technique.

#### 3.2.2. Subcritical Drying

Subcritical drying of solvogels or aquagels is a recognized technique, albeit one employed with varying interpretations. It can be conveniently executed using cost-effective equipment at or near atmospheric pressure, or with pressures and temperatures slightly below the critical point. A shared characteristic across all variations is the wet gels’ aging, followed by a solvent exchange step. The drying process takes several hours to a day or two, making subcritical drying comparable in time requirement to freeze drying. When ambient pressure drying is conducted, the resulting solid material can manifest as either an aerogel or a xerogel, contingent upon the solvent and the gel material’s polarity. As an instance, sol–gel-synthesized silica monoliths with chemically modified hydrophobic surfaces can be subjected to the spring-back effect to yield aerogels [50].

The range of conditions and the quality of the dried material depends on the nature of the solvent that fills the pores, as was studied by Kirkbir and coworkers in making aerogels from atmospheric to supercritical conditions [51]. Shrinkage can be minimized to a few percentages under high-pressure conditions. Lower pressures result in higher shrinkage, which can be extensive at around the atmospheric pressure, as found by Singh and coworkers in making microsphere-based scaffolds for cartilage tissue regeneration [59]. In that situation, the dried product can be considered more a xerogel than an aerogel, but fairly frequently, it is also called an aerogel. The porosity of the low-pressure dried materials is well under or near 90%, compared to the 95–99% porosity of the supercritically or higher-pressure subcritically dried materials. The shrinkage itself is not necessarily a disadvantage. In some instances, it is a desirable feature to increase the stiffness. Subcritical CO_2_ drying was applied to make polymeric microparticles for cartilage engineering by Bhamidipati and coworkers, for example [60].

Subcritical drying was applied by Vazhayal et al. during the synthesis of hierarchically porous aluminosiloxane particles in a sol–gel emulsion process, which was tested as a drug carrier and as an osteoconductive support matrix material for bone tissue engineering. The particles were dried from isopropanol at 50 °C under ambient pressure [61].

#### 3.2.3. Supercritical Carbon Dioxide Drying

Supercritical carbon dioxide drying is one of the most widely used techniques to make aerogel materials. It was used in many cases; thus, only a few examples are listed here. The temperature range is approximately 40 to 80–90 °C, and the pressure range is 75–250 bar. This technique was used for the preparation of a wide range of aerogel materials including chitosan-GPTMS by Reyes-Peces et al. [62], collagen–alginate by Muñoz-Ruíz et al. [63], alginate–lignin by Quaraishi et al. [64], alginate by Martins et al. [65], starch and polycaprolactone by Goimil et al. [45], and silica-TCP-HAp by Lázár et al. [38].

### 3.3. Post-Drying Workup and Shaping

**Figure 2:**
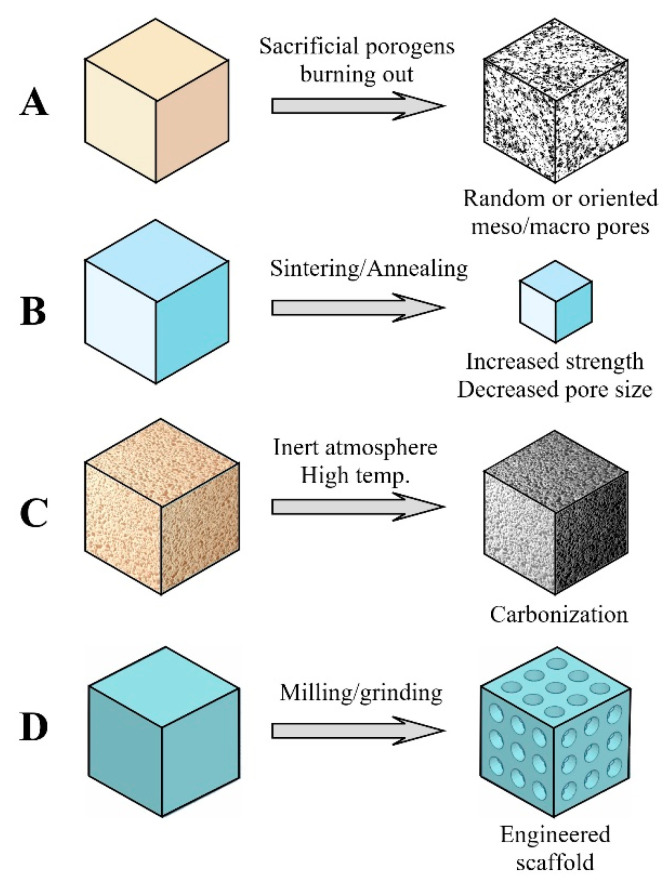
Major types of post-drying thermal or mechanical treatments of aerogels to generate application-specific properties. (**A**) Burning-out sacrificial porogen materials to provide macropores, (**B**) annealing of inorganic aerogels to provide increased mechanical strength, (**C**) high-temperature inert atmosphere carbonization of organic materials to change surface properties, (**D**) mechanical shaping (turning, drilling, milling) to make customized scaffolds.

After the drying process is finished, aerogel materials frequently require further workup, i.e., cutting, mechanical shaping, or thermal treatment to meet the application-specific requirements. The most commonly used techniques are graphically summarized in Figure 2.

Due to the sensitivity of the fine aerogel structure to any kind of wetting liquids, solid-phase post-drying procedures can be used in most cases. Solution-phase soaking, leaching, wet grinding, and melting techniques cannot be applied when the original structure is to be maintained.

Heat treatment is a simple and convenient way to change the porosity, mechanical strength, dissolvation, and degradation properties of inorganic aerogel-based materials. The process is viable only for thermally stable materials like silica, alumina, TCP, and HAp. Meso- and macropores are generated randomly or pre-arranged by burning out sacrificial porogen template materials at a temperature of a few hundred degrees Celsius (Figure 2A). A further increase in the temperature results in some degree of shrinking of the materials. That may increase the compressive strength and hardness to a high level, decrease the pore diameters, and reduce specific surface areas. Silica aerogel-based TCP composites containing the sacrificial porogen material microcrystalline cellulose or ashless filter paper or highly purified cotton fabric heated in the range of 500–1000 °C preserved their mesoporosity (average pore size: 26–46 nm) along with a decrease in specific surface area (from 400 to 184 m^2^/g) and a significant increase in compressive strength (from 0.47 to 16 MPa). Biological activities of the heat-treated materials showed the maximum at 800 °C in cell studies and rat critical size calvaria defect model experiments. The preparation and biological activities of the heat-treated materials were presented by Szabó et al., Hegedűs et al., Kuttor et al., Lázár et al., and Hegedűs and coworkers (Figure 2B) [66,67,68,69,70].

Thermosetting polymer-based aerogel materials (resorcinol–formaldehyde, and polybenzoxazine) alone or in composites with other thermally stable material (i.e., TCP) may undergo an inert atmosphere thermal decomposition and carbonization process at a temperature near 1000 °C. The resulting carbon aerogel materials proved to be biocompatible and supported the growth of human osteoblast cells, as pointed out by Dong et al. and Rubinstein and coworkers [19,71] (Figure 2C).

High-mechanical-strength materials like heat-treated silica-TCP aerogel composites, successfully used in artificial bone substitution in vivo in animal models, can be implanted in load-bearing positions. A high number of applications, however, would require customized mechanical shaping of the specimens to fit in the shape of the defect. Machining, milling, and drilling can be performed with mechanically sufficiently strong materials to provide custom-shaped scaffolds. Silica-HAp or TCP composites, for example, can be drilled and shaped to provide 200–500 micron highly oriented channels that are expected to support bone ingrowth and vascularization in cortical bones [70] (Figure 2D).

## 4. Biomechanical, In Vitro and In Vivo Properties, Toxicity and Biocompatibility

The concept of “biocompatibility”, outlined in 1986 as the “ability of a material to function with a suitable response within a given application”, has remained unaltered and was reaffirmed during the 2018 Consensus Conference in Chengdu, organized under the auspices of International Union of Societies for Biomaterials Science & Engineering [72].

The assessment of biocompatibility encompasses two fundamental criteria: the absence of toxicity and the seamless integration of the material into the biological system. The latter implies that the material should not hinder cellular function and should possess mechanical, chemical, and physical attributes compatible with the facilitation of cell-specific functions [73]. The evolution of artificial bone substitute materials has followed a comprehensive path, traversing through various material generations, each with its distinct attributes and complexities, as comprehensively reviewed by Bongia et al. within the existing scholarly discourse [74].

Conversely, the history of aerogel-based materials is comparatively succinct. Nonetheless, their introduction to the domain introduces an array of distinctive benefits, primarily arising from their intricately porous architectures, which evoke specific tissue responses. The methodologies and protocols governing the scrutiny of biocompatibility and bioactivity commence with meticulously tailored inorganic solutions and culminate in intricate investigations involving living animal models [74].

### 4.1. Biomechanical Properties

The biomechanical properties of different bones and cartilage are well known for quite a long time [75]. Standardized experimental protocols and a wide range of instrumental techniques are used for their characterization. When aerogels are tested, some of the methods have to be significantly modified due to the much lower strength of aerogels. The aerogel-based materials and their scaffolds should match the mechanical properties of the connecting tissues to provide a cooperating and supportive medium for tissue ingrowth, provided the material is implanted in load-bearing positions. That task can be achieved with annealed aerogel-based bioceramic materials [67,68,69,70,76], while other aerogels should be placed in non-load-bearing positions. Most aerogel-based scaffolds contain one or more natural or synthetic polymeric components with or without inorganic counterparts like silica, graphene, carbon nanotube, calcium phosphates, etc. Although the physical properties are important in bone-related research, only a part of the papers contain relevant data [24,42,58,77,78,79,80,81,82,83,84,85,86,87]. Most recently, a critical review paper has been published describing and summarizing the syntheses, biomechanical properties, and their connection with the porosities of bone substitute aerogel materials by Souto-Lopez and coworkers [88].

The most frequently determined mechanical properties of aerogels are the following: compressive strength, Young’s modulus, tensile strength, elastic modulus, stiffness, and shape recovery rate. Although the bone hardness scales (i.e., Vickers hardness, and Shore D) are essential indicators of bone quality [89], they are less frequently used for aerogels. Specialized measurement techniques for this family of materials are described in several papers. Many of them are traditionally related to silica hybrids and composites [90,91]; others deal with elastic organic aerogels [92,93].

### 4.2. In Vitro Testing Methods

The evaluation of artificial bone substitute materials through in vitro testing primarily encompasses distinct categories of assessments. Estimating the toxicity is always a vital step in determining the basic potential of a new preparation. Using diverse cell lines and cell types in controlled cultures is a standard way to assess various parameters, including viability, toxicity, potential immune reactions, adhesion, proliferation, and other pertinent characteristics. Additionally, an important part of the in vitro testing of artificial bone substitute materials revolves around the observation and characterization of the surface hydroxyapatite layer formation in diverse solutions termed as simulated body fluids (SBFs).

#### 4.2.1. Biocompatibility, Cell Viability

Introducing an exogenous aerogel material into a living organism may induce more or less severe immune and inflammatory responses controlled by cytokines. While an initial inflammation of the damaged bone tissue is necessary for collecting osteoprogenitor cells, extended inflammation has adverse effects. Measuring the concentration of the cytokines may provide crucial information on the bone tissue compatibility of the materials [94].

Several methods based on the use of different living cells have been developed to test artificial bone substitute materials. Under controlled conditions, the tested materials are incubated with the selected cells, and from the immune response to the osteogenic differentiation, the studies follow several activities to determine the tested materials’ toxicity, biocompatibility, and bioactivity. Przekora summarizes such examinations in a recent review paper [95].

Cell viability can be determined in the simplest cases by spectrophotometry absorbance measurements or by calculating the ratio of live and dead cells after specific staining and simple optical microscopy plus cell counting. Fluorescent dyes combined with fluorescence spectroscopy or computerized image analysis software may provide information on the number of live or dead cells and cellular activities [96,97]. Cell viability studies are so common that approximately half of the aerogel-related papers are involved; therefore, they are not listed here individually.

#### 4.2.2. Antimicrobial Activity

In general, antimicrobial activity is not an expectation for artificial bone replacement materials. Still, its presence can be beneficial from the point of view of the use of the product. Only chitosan has inherent antimicrobial activity among the components of aerogels prepared for this purpose [98]. Other biopolymers, such as cellulose, gelatin, dextran, pectin, etc., are inactive but suitable for carrying selected antibiotics or gold, silver, platinum, TiO_2_, or ZnO nanoparticles, all possessing antimicrobial activity [98,99,100,101,102].

#### 4.2.3. Simulated Body Fluids

The concept of simulated body fluids finds widespread utilization in the exploration of bioactivity during bone mineralization processes. This testing methodology involves the immersion of samples in clear solutions containing the principal inorganic constituents found in human blood plasma over a span of several days. The inception of this technique traces back to the work of Kokubo and colleagues, who introduced the initial simulated body fluid (SBF) for such investigations [103]. Subsequently, a second publication accentuated the value of these tests [104] in approximating the in vivo bioactivity of distinct categories of bone substitute materials. Several works have effectively extended the application of Kokubo’s test to aerogel-based bone substitute materials [62,66,77,105]. Expanding on the original formulation, researchers have sought modifications to more accurately mirror the comprehensive chemical composition of human blood plasma. Müller et al. ventured to vary the concentration of bicarbonate ions [106], whereas Győri et al. introduced amino acids and serum albumin to generate modified SBFs, thus rendering them more representative of in vivo conditions [107]. Practical considerations in the preparation and application of SBFs encompass crucial steps to prevent precipitation through proper component dissolution sequencing, an approach detailed by Kokubo et al. [104]. Further variations include adopting saturated stock solutions as recommended by Müller et al. [106], or devising a dual-component set of solutions as elucidated by Győri et al. and Vallés Lluch et al. [107,108], thereby simplifying the making of the SBF solution. Achieving a final pH of 7.4 is imperative, while the temperature must remain constant within the 36–37 °C range during the entire course of treatment. To mitigate the risk of bacterial contamination stemming from the presence of glucose, amino acids, or albumin, it is a prudent practice to supplement the SBFs with sodium azide or antibiotics like gentamycin or kanamycin [106,107]. Given the dynamic nature of the bicarbonate/carbon dioxide equilibrium dominant in unsealed containers, the composition and pH of SBFs might fluctuate over time. As a result, experimentation should be confined to sealed vessels, and periodic replenishment of the SBF solution with fresh aliquots becomes imperative. While SBF testing offers a straightforward avenue for estimating the potential for bone formation in artificial bone substitute materials, cautious interpretation is warranted due to the aforementioned intricacies. It is advisable not to exclusively rely on the outcomes of these tests in categorizing or sorting out materials [107].

#### 4.2.4. In Vitro Cell Studies

In the realm of in vitro examinations, a significant portion of research entails the application of diverse cell lines as a fundamental approach. These cell-based investigations offer crucial insights into a range of parameters including cytotoxicity, inflammatory response, cellular metabolism, adhesion, proliferation, and an array of pertinent characteristics of the materials under scrutiny. However, it is important to note that individual cell culture tests may provide insights into only specific aspects of material behavior, with the broader context of intricate tissue reactions or the foreign body response remaining beyond their scope. Despite this limitation, such assays present a convenient and economical means of analysis, exempt from the complexities of permissions and ethical considerations, and are extensively reviewed in the literature [73,109,110]. The cornerstone of these investigations resides in human cell lines, which serve as a fundamental test for gauging and predicting the interactions of the materials in question. However, it is pertinent to acknowledge that access to primary human osteoblast cells remains limited. Consequently, alternative animal cell models have been adopted in the studies. This review does not aim to cover all the tests and protocols; a detailed account of non-aerogel-related areas is summarized in the literature [111,112,113,114,115].

In laboratory studies, a variety of bone tissue cells from both humans and animals are employed to assess cell adhesion, viability, and growth. Key human cell lines include primary osteosarcoma cell lines, such as SaOs-2 and MG-63, which are immortalized and malignant cells. Among non-human cell lines, there are immortalized osteoblast precursor MC3T3-E1 cells from mouse calvaria, primary osteoblast cells from animals like rats, mice, bovines, and rabbits [111], and induced osteoblasts from stem cells of different animal species or humans [115,116]. During these experiments, the focus is on examining cell attachment to surfaces, their viability and proliferation, as well as the potential of stem cells to transform into osteoblasts and the detection of specific indicators of bone metabolism [117].

Tang et al., for example, undertook an investigation involving a 3D-printed nanocellulose/PEGDA aerogel scaffold in conjunction with mouse bone marrow mesenchymal stem cells. Their study revealed that the scaffold exhibited supportive attributes for cell growth, stem cell proliferation, and chondrogenic induction, with outcomes influenced by the Poisson’s ratio sign [52]. In a similar vein, Ge and colleagues crafted a silica aerogel-PCL composite, subjecting it to assessment with MC3T3 and primary mouse osteoblast cells. Their findings demonstrated that the silica aerogel contributed to heightened cell survival, attachment, and growth, while concurrently mitigating the cytotoxicity of the PCL film during prolonged contact [118]. Moreover, Bahrami et al. synthesized collagen aerogel scaffolds coated with reduced graphene oxide (rGO) using a combination of 3D printing and chemical crosslinking, followed by freeze drying. The scaffolds underwent evaluation for bioactivity and bone regeneration potential in both in vitro and in vivo settings. The incorporation of the rGO layer increased mechanical strength by a factor of 2.8 and did not lead to augmented cytotoxicity. Human mesenchymal stem cells displayed heightened viability and proliferation on the scaffold surface. When implanted into cranial bone defects in rabbits, the scaffolds exhibited enhanced bone formation after a 12-week observation period [53].

### 4.3. In Vivo Animal Testing Methods

#### 4.3.1. General Considerations

Broadly, in vitro testing methods involve the utilization of diverse species possessing varying degrees of bone regeneration potential. These assessments encompass scenarios where bone substitute materials are either subjected to soft tissues without direct bone contact (heterotopic testing) or placed in direct proximity to bone tissue (orthotopic testing). These investigations, conducted across different mammalian species, yield insights into immunological responses, histochemical attributes, and cell regulatory mechanisms. By placing materials within artificial defects, the progression of bone remodeling and regeneration is monitored, spanning several months and occasionally extending beyond a year. The bone healing trajectory traverses three principal phases: the sterile inflammatory phase, the repair phase, and the remodeling phase.

#### 4.3.2. The Role of Porosity

The porosity of materials emerges as a pivotal determinant in shaping the in vivo behavior of bioactive substances. Open-pore architectures stand as a remarkably effective conduit for facilitating the transport of materials to and from living tissues, ushering in vital elements like nutrients, oxygen, and signaling molecules. The significance of macroporosity has been expounded upon in the preceding section, elucidating its role in facilitating optimal bone ingrowth. A recent observation by Ratner underscores the role of material porosity in the early stages of regeneration. In instances where identical artificial materials are employed, densely compacted solid structures tend to trigger an inflammatory response in surrounding tissues, characterized as a foreign body reaction. In contrast, porous architectures tend to mitigate the occurrence of such an inflammatory phase [119]. Further insights, such as those offered by Matsiko et al., underscore that scaffold pore size significantly influences the differentiation process of stem cells [9].

The role of porosity of aerogel-based scaffolds has yet to be systematically studied. The need for large pores is well-known in bone tissue ingrowth [120]. However, besides providing a penetrable material-transport channel, the biological role of the much finer aerogel mesopores has yet to be discovered [88]. Comparative cellular studies with chemically identical aerogel samples exhibiting different narrow pore size distribution peaks walking through the entire mesopore and lower macropore region would be desirable to answer the questions.

Foreseen as instigators of minimal foreign body reactions upon implantation, the ab ovo porous aerogel-based materials may hold substantial promise in this context. The intricate structure of these scaffolds can potentially augment this advantage. Collectively, these observations underscore the pivotal role and potential of bioactive aerogel-based materials in the domain of artificial bone substitution.

#### 4.3.3. Selection of the Animal Species

Animal models represent a cornerstone in the exploration of biocompatibility and regenerative potential, both for established commercial and novel experimental artificial bone substitute materials. This paradigm has also been embraced in the assessment of aerogel-based materials. Among the diverse array of animal species, including mouse, rat, rabbit, sheep, dog, goat, and pig, that have been employed in these inquiries, a comprehensive review of their usage, contexts, considerations, and outcomes is available within the literature [121,122,123]. In these experimental investigations, small laboratory animals, mostly rodents, are frequently used due to their accessibility within orthopedic surgical research facilities. However, it is worth noting that their inherent regenerative capabilities may significantly diverge from those of large animals. Although mature large-bodied animals exhibit bone structures akin to humans, their practical availability, expenses, and material demands introduce limiting constraints. The careful selection of the appropriate animal species for experimentation becomes crucial and hinges upon the specific objectives of the research endeavor [124].

#### 4.3.4. Critical and Non-Critical Size Models

The dimensions and location of the bone defect assume a pivotal importance when gauging biological activity. Divergent compositions, structures, and qualities of bones across distinct animal species necessitate the careful selection of the right animal model for evaluating regenerative potentials, as comprehensively covered in the literature [123,124,125,126,127]. The size of the defect bears paramount significance in appraising regenerative capabilities. When working with bones, a defect that lacks spontaneous self-healing throughout the anticipated lifespan of the animal is classified as a critical size defect [69,127]. Contrarily, subcritical size defects may exhibit spontaneous healing. The interposition of the regenerative process with artificial bone substitute materials in critical-size models effectively demonstrates the regenerative potential inherent in the experimental materials. Typically, a single material is evaluated per animal, involving one or two defects. However, there are instances where multiple materials are concurrently tested within the same experimental animal [121].

The materials can undergo testing in load-bearing and non-load-bearing positions. A notably prevalent model in studies involving small animals encompasses the critical-size calvarial defect model. This model expedites material testing in an easily attainable and reproducible manner, obviating the need for precise positioning of experimental materials. In this approach, a disc-shaped sample is nestled within a circular opening atop the cranial bone (typically 6 or 8 mm in diameter), establishing contact with the native bone tissue. An instance of this technique involved the application of a calcium phosphate–silica aerogel composite in rats [67]. While this model is convenient, it does not furnish insights into the functional behavior of the materials, such as their mechanical properties. For investigations of such nature, a load-bearing defect position, such as within the femur, is selected to study the healing and remodeling dynamics of an aerogel material [66].

## 5. Aerogel-Based Materials and Structures for Bone Tissue Engineering

By the traditional IUPAC Gold Book definition, aerogel is a “gel comprised of a microporous solid in which the dispersed phase is a gas”. A problem with the definition is that it does not follow the IUPAC definition of micropores. Aerogels are mostly mesoporous materials containing macropores in some cases. A large portion of aerogels does not have micropores at all. Besides the definition by Hüsing and Schubert, as mentioned in Section 1 [30], the aerogel definition needed fine-tuning. Following the most recent trends supported by several publications, an even broader definition of aerogels, which includes, i.e., the nanofibrous materials as they are appearing in the literature, is applied in this paper. According to the recommendations of Vareda et al. and García-Gonzalez et al., here we use the definition of aerogels as “solid, lightweight and coherent open porous networks of loosely packed, bonded particles or nanoscale fibers, obtained from a gel following the removal of the pore fluid without significant structural modification” [128,129].

Considering the materials and techniques used in bone and cartilage tissue engineering, wet gels, and aerogels have common roots and significant overlapping in many aspects. In this review, we focus only on dry aerogel materials. Independently from their features, only the gels that were dried to aerogels or cryogels by any means will be referred here.

### 5.1. Building Materials of Aerogels and Their Scaffolds Used in Hard Tissue Engineering

The majority of aerogels assessed for their potential in bone regeneration have been constructed from the same building materials widely employed and exhaustively investigated in practical applications. A significant proportion of the tested substances hold approval from the FDA for human usage. The most important characteristics of the materials utilized in the context of aerogel-based bone substitute materials, accompanied by references from the existing literature, are summarized in Table 1.

### 5.2. Aerogel-Based Materials for Bone Substitution

**Figure 3:**
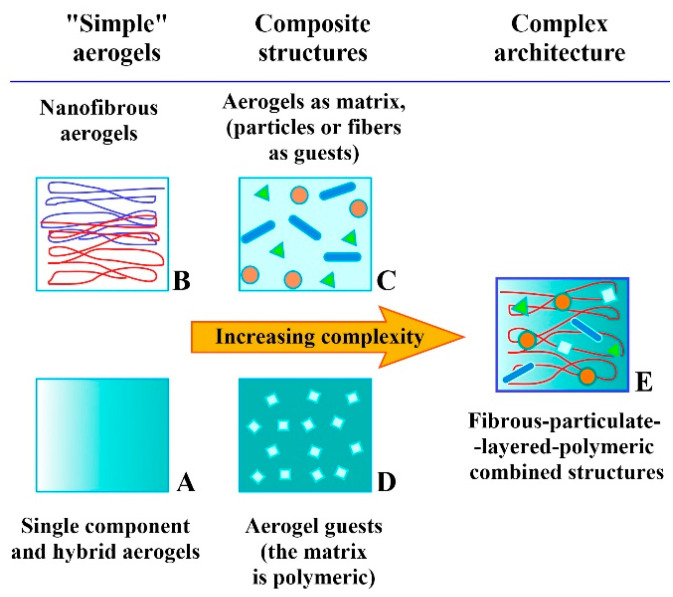
Schematic representation of the major types of chemical composition and structure of aerogel-based artificial bone substitute materials. (**A**) Homogeneous aerogels structures made of single- or multi-component material [15,17,42,61,62,64,65,71,77]. (**B**) Nanofibrous materials dried to an aerogel structure [52,55,56,57,79,190]. (**C**) Aerogel matrix material containing guest particles and/or fibers [14,18,19,53,63,66,67,68,69,70,80,191]). (**D**) Polymeric matrices containing guest aerogel particles [45,46,118,192]. (**E**) Highly complex structures made of aerogels, particles and nanofibers [13,24,27,41,54]. (The different symbols in the figure represent guest particles without further specification.)

Given the wide range and diverse compositions of aerogel-based materials utilized in hard-tissue engineering, a systematic classification based on shared properties becomes necessary. The approach adopted here involves categorizing all aerogel-containing structures, except for single-phase aerogels, as composite materials, characterized by distinct physical phase boundaries. This classification proves especially relevant when natural or synthetic polymeric materials, and complex or layered structures are present. While the chemical composition remains the primary determinant, other factors, such as biocompatibility, bioactivity, cellular responses, and tissue reactions, and other parameters are deterministic and discussed in the previous sections. Pore structures, their multi-dimensional orientation, and the arrangement of different scaffold layers also exert significant influence. Table 1 presents the wide array of chemical components utilized in the field of aerogel-based tissue engineering. Their combination can yield numerous materials, the management of which is not always straightforward. Figure 3 provides an illustrative representation of potential classes and their interconnections, delineating increasing complexities.

A single-phase homogeneous material may be a chemically one-component pristine aerogel or a multi-component hybrid aerogel in which the components are mixed at the molecular level. In that meaning, there is no difference between aerogels of organic or inorganic origin (Figure 3A). Nanofibrous materials from mostly polymeric materials may also be distributed evenly in space by different treatments, forming a gel from which homogeneous aerogels are made by different drying techniques (Figure 3B). Such a homogeneous aerogel phase may serve as the matrix material in which guest particles are distributed (Figure 3C). Polymeric materials may also be combined or fortified with aerogel particles as guests to improve properties (Figure 3D). And finally, all the structures may be evolved into a very complex unit where the matrix and guest functions are combined, and new properties may appear due to the synergistic interaction of materials in the living environment.

**Figure 4:**
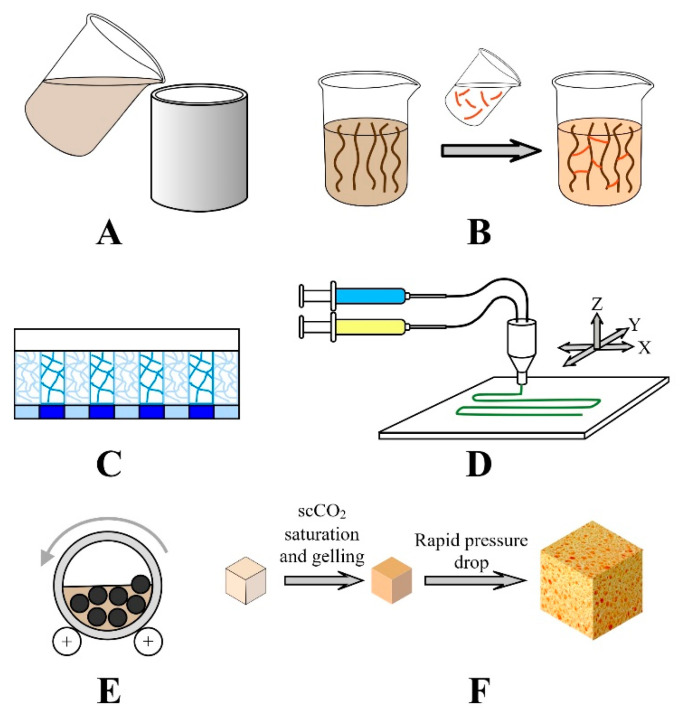
Major types of gel-making techniques leading to simple- or complex-shaped materials prior to drying. (**A**) Sol–gel process and gel casting, (**B**) gelling and crosslinking, (**C**) cryotemplating and freeze casting, (**D**) stereolithography and 3D printing, (**E**) ball milling, (**F**) supercritical CO_2_ gelation and foaming. (The gel is made with scCO_2_ then expanded by a rapid pressure drop).

The way aerogels are made for hard-tissue-engineering purposes depends on the material and the properties of the aerogel phase, as well as the complexity of the structure. However, independently from the nature of the materials and the final complexity of the structures, the common point is that all “pre-aerogels” go through a wet gel state, from which the final aerogel is prepared by a suitable drying technique. The technical implementation of wet gel-making procedures is summarized and shown in Figure 4. The simplest and most traditional way, as mentioned in Section 2, is the sol–gel technique (Figure 1).

In the gel-casting process (Figure 4A), the reaction mixture is poured into a mold and allowed to set there. The casting process may be combined with the addition of guest particles, fibers, or nanofibers, followed by crosslinking chemical reactions (Figure 4B). Freeze casting is the way to make controlled bimodal pore size distribution by programmed zone freezing of the solvent in the gelation phase (Figure 4C). Stereolithography processes use chemical crosslinking or photochemical polymerization in special 3D-printing techniques to provide custom shape and geometry of scaffolds with controlled macroporosity (Figure 4D). Nanofibrous gels are made from natural nanofibers or electrospun mats by ball milling in an adequately selected solvent (Figure 4E). A rarely used technique is the supercritical gelation and foaming initiated by a rapid pressure drop of gas-saturated polymeric materials combined with other gel-making steps (Figure 4F).

#### 5.2.1. Single-Component and Hybrid Aerogels

Creating biocompatible aerogels for hard-tissue replacement can be achieved through a straightforward approach. One option involves using a single biocompatible or bioactive component, or alternatively, combining multiple such ingredients to form a hybrid structure without macroscopic or micron-level internal phase boundaries. Subsequently, these gels can be dried to aerogels without encountering any constraints in the drying process. This custom formulation allows for the development of the essential macroporous structure crucial for facilitating optimal bone tissue ingrowth.

Silica–chitosan hybrid aerogels were synthesized by Perez-Moreno and coworkers in a sol–gel process from TEOS and chitosan with the help of high-power ultrasound. Chitosan improved the mechanical properties of the gels, which were dried with supercritical CO_2_ to monoliths with very high specific surface area (786–1072 m^2^/g), and a 0.13–0.20 g/cm^3^ density range. The aerogels were tested in simulated body fluid, and found that the surface silanol groups promoted the nucleation and formation of hydroxyapatite crystals on the surface, which is an indication of bioactivity. Human osteoblast cells were cultured on the aerogel surface and immunolabeled to monitor cytoskeletal changes and focal adhesion. The aerogels proved to be osteoconductive and osteoinductive in the cell studies [77].

Maleki et al. synthesized a silica–silk fibroin hybrid aerogel scaffold with honeycomb micromorphology and multiscale porosity manufactured from TEOS and silk fibroin in the presence of hexadecyltrimethylammonium bromide in a one-pot acetic acid-catalyzed sol–gel reaction and unidirectional freeze casting, which controlled the size of the macropores in the ten-micron range. The reason for the combination of silica and silk fibroin was to increase the pore size regime and the mechanical strength synergistically. Mechanical strength increased to a 4–7 MPa Young’s modulus. The as-prepared aerogel proved to be cytocompatible and nonhemolytic, showed no toxicity, and triggered MG63 osteoblast cell attachment and proliferation in 14 days. Implantation of the material in rat femur bone defects resulted in bone formation in 25 days [42].

Polybenzoxazine (PBO) aerogel and its hybrid with resorcinol–formaldehyde (PBO-RF) were prepared and then carbonized at high temperature by Rubenstein and coworkers. Human calvarial osteoblasts were used in the biological studies. Results showed that PBO aerogel and its combination with RF and the carbonized aerogels are compatible with the osteoblasts. However, PBO-RF aerogel resulted in a low growth rate of cells. Carbonized PBO aerogel had better mechanical properties and high porosity. It proved to be the most advantageous for osteoblast growing, which makes the material a promising candidate for tissue-engineering applications [71].

Horvat and coworkers prepared a methoxyl pectin–xanthan aerogel layer on the surface of medical grade stainless steel from the aqueous solution of high-methoxyl pectin and xanthan in an optimized 1:1 ratio by an absolute ethanol-induced gelation process, after which the gel layer was dried with a continuous flow of supercritical CO_2_. Non-steroidal anti-inflammatory drugs diclofenac sodium and indomethacin were loaded in the aerogel either from the saccharide solution directly or from an ethanol solution in the soaking phase. After drying, their release profiles were determined. The aerogel layer protected the steel surface from corrosion, and the loaded drugs were released in one day. The biocompatibility of the layer material was tested after dissolving the aerogels in a buffer with a human bone-derived osteoblast hFOB cell line. The results showed higher viability and better proliferation of the cells in the aerogel solutions than in the control samples [15].

Quraishi and coworkers prepared meso–macro porous alginate–lignin hybrid aerogels from a basic solution of alginate and lignin, containing calcium carbonate particles gelled under a CO_2_ atmosphere (45 bar for 24 h), then foamed by a controlled release of pressure. The as-prepared gels were subjected to solvent exchange and then CO_2_ supercritical drying. The biocompatibility of the materials was tested using a mouse fibroblast-like cell line L929. The aerogels proved to be non-cytotoxic in cell studies compared to tissue culture polystyrene reference. The cell viability was similar to that of the control, and the materials showed good cell adhesion and indicated no negative effect of the lignin component. The alginate–lignin aerogels are good candidates as scaffold materials for further in vivo tissue-engineering studies [64].

Calcium– alginate also served as one of the major components in new alginate–starch aerogels prepared by Martins et al. The wet gels were made from an aqueous solution of sodium alginate and starch in the presence of calcium carbonate particles. The gelation occurred under the acidification effect of high-pressure carbon dioxide. A rapid release of carbon dioxide produced a foamed material that was then dehydrated with anhydrous ethanol and dried to aerogel with supercritical carbon dioxide. The macropore formation sharply depended on the rate of depressurization. In simulated body fluid, the material developed surface hydroxyapatite crystals indicating bioactivity potential, which was attributed to the presence of calcium ions. Cell studies with fibroblast-like cell line L929 showed no cytotoxic effect, and the cells colonized the surface. Thus, the alginate–starch hybrid material may be applied in biomedical research and bone repair [65].

Vazhayal and coworkers synthesized mesochanneled and tunable bimodal pore size distribution aluminosiloxane microspheres from acidic pre-hydrolyzed aluminum isopropoxide sol stabilized with PVA and aminopropyl trimethoxysilane solution injected in ammoniac paraffin oil that initiated self-assembly and solidified the droplets. After fortification of the structure by soaking in TEOS, the microspheres were washed, solvent exchanged, and dried to aerogel under subcritical conditions at 50 °C and ambient pressure. Finally, the microparticles were calcined at 600 °C to provide a pH-responsive, controlled-release drug carrier material. NSAIDs were adsorbed in the aerogel from hexane solutions, and the preparations were tested for release in simulated gastric and intestinal fluids. The biocompatibility and cytotoxicity of the aerogels were tested in vitro on normal H9c2 cells, while gastric ulceration was tested in vivo on albino male rats. Although it was not tested directly, the authors envisaged utilizing these aerogel microspheres in potential bone tissue engineering [61].

To defeat the mechanical limitations of the traditional hydroxyapatite scaffolds, a new highly porous and elastic single-phase aerogel material made from hydrothermally synthesized and freeze-dried ultra-long hydroxyapatite nanowires was prepared by Huang and coworkers. The biological activity was tested with rat bone marrow mesenchymal stem cells. The results showed that the material promotes cell adhesion, proliferation, and migration of the cells and elevate the expression of osteogenesis- and angiogenesis-related genes. The nanowire aerogel scaffold can promote the ingrowth of the new bone and neovascularization in the bone defect region, thus making this a promising material for bone tissue engineering [17].

Osorio and coworkers made sulfate or phosphate half-ester-functionalized cellulose nanocrystals and crosslinked them through the carboxylate derivative with adipic acid dihydrazide. The as-prepared materials were transformed into cryogel by freezing in molds at −5 °C, and then ice crystals were removed by soaking in absolute ethanol. Finally, the materials were dried with supercritical CO_2_ to aerogels. The bioactivity was tested on SaOS-2 cells, and the materials showed an increase in cell metabolism for seven days. A simulated body fluid test showed hydroxyapatite layer formation after the materials were pre-treated with calcium chloride solution. The sulfated aerogel proved to be more advantageous regarding mechanical strength and stability under an aqueous environment. In vivo implantation in the calvaria of male rats showed a significant increase in bioactivity in 12 weeks, proving that the new and flexible materials can facilitate bone ingrowth [13].

Reyes-Peces et al. combined chitosan with hydrolyzing 3-glycidoxypropyl-trimethoxysilane (GPTMS) in an acid-catalyzed sol–gel process at 50 °C followed by supercritical CO_2_ drying resulting in a mechanically exceptionally strong aerogel material. The crosslinking with GPTMS connects the amino and hydroxyl groups of the polysaccharide chains into a hybrid interconnected silica plus carbohydrate network. In vitro, biocompatibilities were proved by the hydroxyapatite layer formation in simulated body fluid. The in vivo bioactivities were tested on human osteoblast cells. No cytotoxicity was observed; the material induced cell adhesion and the cells showed cytoskeletal rearrangements and elongation with stress fibers [62].

#### 5.2.2. Nanofiber Aerogels

Electrospun PLGA-collagen-gelatin nanofibers combined with Sr-Cu co-doped bioglass fibers and bone morphogenetic protein 2 (BMP-2) were combined in a 3D hybrid nanofiber aerogel network by Weng et al. The new material was tested for cranial bone healing using the critical-size rat calvaria model. The sustained slow-release of BMP-2 proteins from the degradable aerogel increased the rate of bone healing significantly and improved the vascularization. Histopathology data showed a near-complete degradation of the aerogel material in the regenerated tissue [79].

Xu and coworkers transformed electrospun polycaprolactone nanofibers into soft, elastic, and very porous aerogel scaffolds by freeze grinding the nanofibers and then by thermally inducing the self-agglomeration, and the as-prepared gels were freeze-dried. In vitro studies with mouse bone marrow mesenchymal stem cells showed high cell viability. Depending on their elasticities, the materials favored osteogenic or chondrogenic differentiation of the stem cells. In vivo experiments indicated that the highly porous and elastic scaffold can act as a favorable synthetic extracellular matrix for bone and cartilage regeneration [193].

Rong and coworkers prepared silk fibroin (SF)–chitosan (CS) aerogel scaffolds rein-forced with different amounts of SF nanofibers (SF-CS/NF1%, SF-CS/NF2% and SF-CS/NF3%) for bone regeneration. In vitro cytotoxicity test against MC3T3-E1 cells con-firmed that all samples were biocompatible while further experiments confirmed that by rougher surface, enhanced mechanical strength and well-regulated pores, this biocompatible scaffold significantly facilitated osteogenic differentiation [194].

#### 5.2.3. Aerogels as Matrix Materials

Silica aerogel–tricalcium phosphate and hydroxyapatite composites were synthesized, and their potential in artificial bone substitution was systematically studied by Szabó et al., Győri et al., Hegedüs et al., Kuttor et al., and Lázár et al. The silica matrix was synthesized in a sol–gel process from TMOS under basic conditions. Microcrystalline or nanocrystalline TCP and/or HAp, which acted as bioactive components, in addition to microcrystalline cellulose, were all dispersed in the reaction mixture in the gelation phase. Large monoliths, small cylinders, spheres, and irregularly shaped particles were prepared and dried with supercritical CO_2_ at 80 °C. Cellulose was a sacrificial porogen material and burned out at 500 °C. High-temperature annealing (in the range of 500–1000 °C) of the samples resulted in a change in their dissolution profile and mechanical strengths, but the mesoporous structure and high specific surface area were preserved at all temperatures. The highest temperature provided the highest rate of shrinkage and also the highest compressive strength (up to 102 MPa). The 900 ° and 1000 °C materials were strong enough to be tested in load-bearing positions. The in vitro SBF examination resulted in microcrystalline HAp layer formation on the surface. The cellular metabolism and proliferation were studied with MG-63 cells, while gene expression studies were also performed on SaOS-2 cells. In vivo small animal studies used 1.5 mm diameter cylinders in rat femurs and 8 mm discs in rat calvaria defect models. Both series of animal experiments proved the bioactivity and bone regeneration potential of the silica aerogel-TCP composites in a few months. The highest bone regeneration potential was observed with the 800 °C temperature sample versions [66,67,68,69,70,107].

Tevlek and coworkers synthesized electrically conductive carbon aerogels decorated with tricalcium phosphate nanocrystallites. The decorated aerogel was made from cellulose fibers, while TCP was also added. Freeze drying produced the pristine aerogels that were heated at 850 °C or 1100 °C under argon atmosphere. The new aerogels were not cytotoxic when tested on P9 L929 mouse fibroblast cells. Proliferation and attachment were tested using disk-shaped specimens with MC3T3-E1 mouse pre-osteoblast cells, providing, thus, a future possibility of applying electric stimuli that might have a significant effect on the cellular behavior [18].

Muñoz-Ruíz and coworkers synthesized a highly porous collagen–alginate aerogel-based scaffold with and without graphene oxide mixed in. The buffered solution of collagen and alginic acid (and graphene oxide) was crosslinked and gelled with calcium chloride, solvent-exchanged with ethanol, and dried under supercritical CO_2_ conditions to form the aerogel. Osteoblast cells seeded on the surface of collagen–alginate aerogel showed adhesion, proliferation, and some degree of extracellular matrix formation after 48 h of incubation. In contrast, the graphene oxide-containing aerogel did not support the cellular growth and activity [63].

Chitosan (CH) matrix was combined with an electrospun nanomaterial of cellulose acetate (CA) and poly(ε-caprolactone) (PCL) by Zhang et al. in a ball-milling process and then freeze-dried to the aerogel CA/PCL/CH. The material showed increased mechanical strength and was bioactive in studies with the MC3T3-E1 cell line. It promoted cell adhesion, infiltration, and osteogenic differentiation [57].

Dong and coworkers prepared beta-tricalcium phosphate-based specimens with printing or compression and soaked them in the premix of a resorcinol–formaldehyde (RF) wet gel. After setting, the samples were dried under ambient conditions and carbonized at high temperature, resulting in the carbon aerogel-coated β-TCP scaffold, which was then used in photothermal therapy. The material was not only effective in ablation of osteosarcoma tumors but promoted osteogenesis as well [19].

A new composite aerogel composed of nano-hydroxyapatite (n-HAp), silk fibroin, and cotton cellulose, crosslinked with epichlorohydrin, and freeze-dried from tert-butanol was developed by Chen et al. to overcome the mechanical problems of the previously synthesized n-HAp biopolymeric composites. Uniaxial compressing of the aerogel showed increased mechanical strength and toughness, making the values similar to that of the cancellous bones. HEK-293T cell studies of the material showed a high ability of cell adhesion, proliferation, and differentiation [58].

Tetik et al. prepared bioinspired aerogels resembling the pore structure of the bones using the unidirectional freeze-casting process followed by freeze drying, resulting in layered mesoporous and macroporous regions. Colloidal silica and graphene oxide were used as base materials. The study focused on the technical aspects of the process. The as-prepared structured aerogels were not tested for bone substitution potential [41].

Graphene oxide (GO) (in the 0–0.2% range) and Type I collagen-containing composite aerogels with enhanced stiffness were prepared by Liu et al. to improve the bone repairing potential of large monolithic aerogel pieces. The aerogel materials were tested in the rat cranial defect model, which proved its biocompatibility and osteogenic activity. The graphene oxide content positively affected the mechanical properties, and the 0.1% GO content produced the highest biological activity [80].

Wu and coworkers synthesized an alginate aerogel combined with in situ-prepared octahedral metallic copper nanocrystals stabilized with carbon dots and loaded with the antibiotic tigecycline. The aerogel proved to be an efficient slow-release antibacterial agent in which the antibiotics and the copper ions acted synergistically. The as-prepared aerogel material showed low cytotoxicity and may be important in preventing bone infections leading to osteomyelitis [14].

Nanoparticles consisting of the miR-26a and a cationic polymeric gene delivery vector (HA–SS–PGEA) were embedded by Li et al. in an electrospun 3D matrix made of poly(lactic-co-glycolic acid) (PLGA)–collagen–gelatin (PCG) and bioactive glass (BG). The scaffold proved to be a promising bone graft candidate in the rat cranial defect model. The molecular mechanism of the mesenchymal stem cells is governed by the microRNAs. In the osteoblastogenesis process, miRNA-26a acts as the promoter of the osteogenic differentiation of bone marrow derived from mesenchymal stem cells [27].

Scaffolds made from type I collagen aerogel (Col) and reduced graphene-oxide–collagen aerogel (Col-rGO) were synthesized by Bahrami et al. in a two-step crosslinking and freeze-drying process. The addition of rGO improved the mechanical strength, and the aerogel showed no cytotoxicity and increased the viability and proliferation of human bone marrow mesenchymal stem cells. The rabbit cranial defect model showed an increased rate of bone formation [53].

#### 5.2.4. Aerogels as Guest Particles

The matrix material polyethylene glycol diacrylate was combined with hydrophilic and highly biocompatible cellulose nanofibrils (PEGDA/CNF) by Sun et al. in different compositions and printed out in a self-built stereolithographic method using a hexagonal mask pattern irradiated with white light, followed by freeze drying to dry aerogel scaffolds. Soaking the aerogels in water resulted in significant water uptake, leading to aerogel–wet gel combo materials. Mechanical properties and the biocompatibility of the as-prepared wet materials were tested. The non-toxic aerogel–wet gel scaffolds were of a porous nature that proved to be advantageous for the adhesion of bone mesenchymal stem cells [55].

PMMA-based bone cements are widely used in the medical practice in filling bone cavities or fixing metallic implants in position. Although such bone cements are bioinert materials, ossification is not induced on their surface. Lázár and coworkers embedded functionalized silica aerogels as guest particles in in situ polymerized PMMA matrix and tested them in simulated body fluids for bioactivity. Results showed that the compressive strengths were increased compared to the neat PMMA. SBF solution resulted in a dissolution of the hydrophilic silica aerogel from the polymeric matrix, leaving a highly porous surface behind. In contrast to the smooth surfaces of the PMMA bone cements, the newly developed porous surface may be advantageous, providing a better bone tissue adherence [192].

Goimil and coworkers embedded starch aerogel microspheres in a supercritically foamed poly(ε-caprolactone) (PCL) highly porous scaffold, increasing the interconnectivity of the pores and the specific surface area. The composite was loaded with ketoprofen under supercritical CO_2_ conditions and showed a sustained ketoprofen release at pH 7.4. Starch aerogel microspheres mildly decreased the mechanical strength and increased the drug release rate compared to the pristine PCL matrix [45].

Silica aerogel was embedded in poly(ε-caprolactone) (PCL) by Ge et al., and its presence prevented any cytotoxic effect of PCL in a long period of time in contact with tissue cultures. It improved the survival and growth of 3T3 cells and primary mouse osteoblast cells. Silica aerogel helped maintain the pH and prevented the acidification of the connecting tissues for four weeks [118].

Silk fibroin aerogel is embedded in supercritically foamed poly(ε-caprolactone) (PCL) scaffolds loaded with dexamethasone under scCO_2_ conditions by Goimil et al. Silk fibroin is a cell-adhesion promoter, while dexamethasone is an osteogenic differentiation agent. The aerogels improved the pore structure and, thus, the biological fluid transport and facilitated the cell infiltration. The in vivo calvarial test showed the importance of the form of dexamethasone, which promoted bone tissue regeneration [46].

Finely ground heat-treated silica aerogel-TCP composites were embedded in PVA/chitosan electrospun nanofiber (147+/−50 nm diameter) meshes and crosslinked with citric acid by Boda and coworkers. Dental pulp stem cells were seeded onto the surfaces and proved the bioactivity of the materials. Rat critical-size calvarial models were used to test the role of the meshes on bone regeneration. After six months, significant new bone formation was observed, proving that the hybrid nanospun scaffolds containing bioactive aerogel guest particles may be used as new experimental bone substitute bioactive materials [195].

#### 5.2.5. Complex Aerogel Structures

Incorporating complex structures defies straightforward categorization. Often, classification appears arbitrary due to varying perspectives. This challenge is particularly evident with composites or scaffolds, where determining whether the aerogel phase serves as the host or the guest becomes intricate, especially within multicomponent systems.

Zhang and coworkers prepared 3D fibrous composite aerogels in a three-layer gradient structure from poly(L-lactide)/gelatin composite fibers, glycosaminoglycan in the top layer, and apatite in the middle and lower layers. The properties of the materials are described in detail in Section 5 [24].

Li et al. prepared strontium ranelate (SR) and incorporated it in mushroom tyrosinase enzyme-induced crosslinked gelatine nanoparticles/silk fibroin gel that was freeze-dried to aerogel. Rapid deposition of HAp on the surface of the scaffold took place, but initial burst release of strontium did not occur. Instead, increased osteogenic differentiation of osteoblasts and inhibiting the activity of osteoclasts was observed in ovariectomized rats using the calvaria defect model [196].

Asha and coworkers made reduced graphene oxide (A-rGO) aerogel from rGO with citric acid at 90 °C in aqueous solution. After gelation, the first aerogel was made by freeze drying. After that, it was functionalized with chitosan by soaking A-rGO in chitosan solution, then freeze-dried again. HAp particle decoration was made by soaking in SBF. MG63 cell studies indicated that the chitosan interfacial layer improves biocompatibility, and the mineralized chitosan layer increased the cell viability and proliferation [54].

Ferreira et al. combined a colloidal aqueous suspension of cellulose nanofibrils (20%) and bioglass particles (80%) in an interconnected 3D network, freeze-dried to a porous cryogel structure. A hydroxyapatite layer was formed on the surface in SBF, shown by the red stain (Alizarin Red) and the IR spectroscopy, indicating good in vitro biocompatibility of the material. The bioglass content provided the necessary ions to facilitate BMP-2 production in cells. The combined material in the in vivo experiments showed no liver or kidney toxicity. Rat calvarial defect experiments proved that the composite material induced new bone formation in 57 days [56].

## 6. Aerogel-Based Materials for Cartilage Tissue Engineering

In comparison to bone substitution, only a limited number of publications address the utilization of aerogel-based materials for cartilage tissue regeneration. This endeavor encounters notable mechanical, chemical, and biological complexities. Articular cartilage comprises four key constituents: type II collagen, proteoglycans (glycosaminoglycans bound to proteins), water, and chondrocyte cells embedded within the extracellular matrix. Unlike bone, articular cartilage lacks intrinsic self-repair capabilities. Consequently, all essential elements must be supplied externally, with material transport occurring gradually through the synovial fluid. Notably, chondrocyte cell density is much higher on or near the gliding surface compared to the base layer, with cellular morphology and orientation varying by depth. Artificial scaffold materials necessitate seeding with chondrogenic cells sourced from the patient or, more recently, utilizing undifferentiated stem cells. Furthermore, maintaining a continuous mechanical stimulus in vitro is crucial to foster the development of a compression and impact-resistant surface characterized by aligned chondrocytes and collagen fibers parallel to the surface [197,198].

Chen et al. made 3D aerogel-like scaffolds from electrospun nanofibers containing gelatin–polylactic acid and gelatine–polylactic acid–hyaluronic acid and studied their bioactivity. In vitro examinations proved the adhesion, growth, and proliferation of chondrocyte cells. The materials were elastic and showed a sort of shape memory effect. The rabbit articular cartilage injury model indicated that gelatin-PLA had only a limited cartilage-repair effect. However, the hyaluronic acid-modified gelatin-PLA scaffold proved to be more active in cartilage regeneration [190].

Scaffold materials with tunable mechanical properties were synthesized by Tang and coworkers from nanocellulose fibers and polyethylene glycol diacrylate (PEGDA) by stereolithography (SLA), and the wet gels were freeze-dried to an aerogel. The macropore sizes were basically determined by the parameters of the SLA process. Mouse bone marrow mesenchymal stem cells showed proliferation and induction, making the material a promising candidate for the cartilage repair [52].

Zhang and coworkers prepared aerogel-based gradient scaffolds to provide an artificial bioactive interface between the bone and the cartilage tissue. Three layers of 3D fibrous aerogel structure were constructed in a gradient arrangement from electrospun poly(L-lactide), gelatin, glycosaminoglycan, and hydroxyapatite. The aerogel layers were prepared separately from electrospun mats by homogenization, freeze drying, and crosslinking with heat, then mineralized and glued together with gelatine. The hierarchical aerogel scaffold induced the bone mesenchymal stromal cells, which differentiated into chondrogenic and osteogenic phenotypes specific to the zone they were in contact with. Cell affinity peptide E7, intended to enhance cell migration, was grafted in the gradient structure by soaking the pre-treated aerogel in its aqueous solution. Aerogel scaffolds without composition gradient and scaffolds with gradient aerogels were implanted in rabbit knees and monitored for 12 weeks for tissue regeneration. The results showed that the gradient aerogels could reconstruct an osteochondral interface, and the E7 peptide-containing aerogel scaffolds are promising candidates in tissue engineering [24].

Three-dimensional porous nanocomposite scaffolds based on cellulose nanofibers for cartilage tissue engineering were prepared by Naseri and coworkers containing freeze-dried cellulose nanofibers as the major component in a gelatin and chitosan matrix crosslinked with genipin. The scaffold showed a macroporous structure of interconnected pores. The dry material’s mechanical strength (compression modulus) was higher than that of the natural cartilage tissue and lowered in phosphate-buffered saline solution. The high porosity and compatibility with the chondrocytes made the material interesting for cell attachment and extracellular matrix production [198].

## 7. Challenges, Opportunities and Future Trends

Bone and cartilage tissue regeneration materials have evolved through three distinct developmental stages. Initially, they served as simple bioinert tissue support (first generation). Subsequently, advancements led to the emergence of nano-engineered resorbable composite materials containing bioactive molecules and growth factors (third generation), as elucidated by Hench and Polak [199]. Now, we look towards the future of this biomedical sector of aerogel research, seeking answers to several key questions. How can the tissue regeneration potential be further enhanced in the fourth generation of materials? How can aerogel-based materials, structures, and devices take advantage on these developments? And how can the new technical extensions be effectively integrated or combined with aerogels?

Aerogel-based bone and cartilage substitution has encountered various challenges from its inception. The use of substances already approved for clinical applications proves invaluable in selecting aerogel building materials with the desired bioactivity. One of the remarkable advantages of aerogels lies in their high porosity and interconnected open-pore structure, which offers additional benefits. The porosity plays a crucial role in enhancing their bioactivity and tissue reactions, facilitating the efficient transport of dissolved oxygen and nutrients to surrounding tissues, and potentially reducing the occurrence of foreign body reactions. Another noteworthy feature is the high specific surface area of aerogels, allowing them to be loaded with bioactive small molecules during the gelation or nanofiber-making stages, or even after drying. Supercritical adsorption is a convenient method for loading aerogels while preserving their original structure. Additionally, during the gelation phase, protein-like molecules, macromolecules, growth factors, and even living cells may be embedded within the aerogel, which can later be freeze-dried.

Improving the benefits of aerogel-based materials could be further refined by crafting oriented, multi-component, and function-specific layered structures or scaffolds, featuring concentration gradients, precisely tailored macropores, and channels that mirror the intended tissue’s architecture for regeneration. The inclusion of oriented macro-channels could facilitate tissue ingrowth and promote vascularization. Various techniques, such as successive casting, electrospinning, stereolithography, 3D printing, selective leaching, cryotemplating, and employing sacrificial porogens, along with post-drying manufacturing, can be employed to achieve these specialized materials. Through the integration of materials within a carefully planned hierarchical structure and the utilization of additive manufacturing techniques, the bioactivity can be heightened. Looking ahead, the potential for improved efficacy in bioactive aerogels may also stem from the discovery of new building materials and unexplored synergistic interactions. Utilizing aerogels made from materials that have already gained approval and clinical licensure may offer the advantage of expediting the approval process for animal experiments. However, the continuous quest for novel materials remains paramount in uncovering novel interactions with living tissues, underscoring the persistent need for innovation in this area.

In addition to their advantages, aerogel-based materials may have some drawbacks when compared to traditional tissue-engineering materials. One significant concern is their mechanical properties, which may not be suitable for load-bearing positions, except for aerogel-based bioceramics. Moreover, the variable sensitivity of aerogel materials to wetting liquids like water, body fluids, or blood can also pose challenges for certain applications. In terms of manufacturing, subcritical and freeze-drying techniques have shown potential for upscaling to economically feasible high volume levels due to their relative simplicity and lower costs. However, supercritical drying, while not impossible, is more difficult and costly to be upscaled to produce large quantities of aerogel-based materials. Despite these limitations, ongoing research and advancements in aerogel technology continue to address these challenges and open up new possibilities for their use in tissue-engineering applications. As the field progresses, we can expect to see further developments to overcome these drawbacks and fully harness the potential of aerogel-based materials in regenerative medicine.

In the foreseeable future, the field of aerogel-based bone and cartilage tissue engineering is poised for ongoing development. The research will continue to create new materials, combine existing ones, and explore synergistic interactions to enhance outcomes. Additionally, there will be a concerted effort to fabricate intricate 3D scaffold structures that closely emulate the composition, hierarchy, and functions of the target tissues.

In the more distant future, the trajectory of aerogel-based tissue-engineering materials appears to be closely linked with the advancement of the fourth generation of bone and cartilage tissue-engineering materials, as described or anticipated by Ning et al. [200]. A potential outcome of future investigations could involve the integration of next-generation aerogel-based scaffolds with implantable and biodegradable power sources, along with microelectronic circuits capable of continuously monitoring the progress of the healing process. While the fundamental components of such electronic devices and power sources have been developed, their incorporation into implants remains an ongoing endeavor. In relation to aerogels, a few promising examples exist that could potentially open new avenues for innovative solutions in the years ahead.

Hong and colleagues have already developed graphene aerogels incorporating electrically conducting polydimethylsiloxane sheets. These materials bear structural and functional resemblance to cartilage tissue found in articular joints. These innovative constructs have found application in sensor technology, capable of transmitting signals concerning mechanical force intensity during joint loading [33].

Another avenue for exploration is the potential to enhance or stimulate bone healing through external stimuli. As demonstrated by Caliogna et al., pulsed electromagnetic fields can initiate or bolster the healing process [201]. Although dedicated aerogel-based composite devices designed to generate or support external stimuli are not yet available, a noteworthy advancement is exemplified by the electrically conductive carbon aerogel adorned with ceramic tricalcium phosphate nanocrystallites, as developed by Tevlek and colleagues. This work could potentially pave the way for upcoming advancements [18].

The existing devices and therapies have already demonstrated certain aspects of the concept. For instance, microwave devices are employed for the sensing the bone-healing process [202], and low-dose microwaves have also been tested to promote bone healing [203,204]. Electrical stimulation has been extensively studied in bone therapy [205], while infrared laser has been found to aid in bone healing when combined with bone morphogenetic protein [206]. Even red visible light has shown potential in promoting bone regeneration [207]. These examples highlight the diverse array of approaches being explored to enhance bone healing and tissue regeneration.

## 8. Conclusions

Aerogel-based materials continue to play a significant and evolving role in orthopedic and dental research. The strategic combination of bioactive inorganic, organic, natural, and synthetic polymeric materials within aerogel matrices has notably enhanced the biocompatibility and bioactivity of engineered bone and cartilage substitutes. Leveraging their exceptional porosity and customizable surface properties, these grafts and scaffolds create a conducive milieu for stem cell growth, proliferation, and differentiation, fostering osteogenic development. In vivo animal studies have underscored that aerogel-based materials exhibit not only biocompatibility but also osteoinductive properties and active bioresorption, leading to the regeneration of deficient bone tissues.

By incorporating aerogels with established bioactive materials, the adverse effects linked to the degradation of polymeric materials have been mitigated. Recent works have yielded highly oriented and layered aerogel architectures that closely emulate the intricacies of living tissue environments. These materials have already demonstrated their potential in healing and regenerating bone and cartilage tissue defects.

In the near future, the refinement of scaffold designs tailored to specific application sites, coupled with novel material combinations, is poised to amplify their therapeutic efficacy and biomedical utility. Beyond advancements in chemical composition and structural intricacy, the next developments of aerogel bone substitute materials may involve external interactions after implantation, to both bolster and monitor the healing process.

## Figures and Tables

**Table 1:** Building block materials used for aerogel-based bone and cartilage tissue engineering. Their bulk bioactivities were tested in vivo, and many of them are used in clinical practice. The references in the table are mainly review papers summarizing the properties, in vivo effects, and therapeutic results achieved in the non-aerogel era.

Name	Properties	References
Alginate	The β-D-mannuronic acid and α-L-guluronic acid-containing alginates can be formulated into gels, particulate solids, nanofibers, or ordered microstructures. They are frequently combined with other biomolecules or chemically modified. Alginates exhibit excellent biocompatibility, biodegradability, and tunable cell-binding affinity, making them versatile materials in wound healing, drug delivery, cartilage, or bone tissue repair.	Sun and Tan; Martau et al. [130,131]
Aluminosilicate	Aluminosilicates show zeolite-like structures and link to the bone matrix. The coating on the alumina surface shows good biocompatibility with the osteoblasts that can sustain their bioactivity.	Oudadesse et al. [132]
Bioactive glass, Bioglass	Bioactive glasses exhibit excellent tissue binding and good bone regeneration properties. Their chemical composition is described with different SiO_2_, Na_2_O, CaO and P_2_O_5_ ratios. Depending on the composition, they may also bind to soft tissues. In combination with other bioactive materials, they are frequently used in bone scaffolds. Silicate ions liberated in the degradation process promote the formation of Type I collagen. Bioactive glasses are FDA-approved bone graft materials.	Bellucci et al.; Gerhardt and Boccaccini [114,133]
Carbon (amorphous, graphitized)	Carbon forms are insoluble and non-resorbable (thus permanent) bioinert materials made by high-temperature carbonization of resorcinol–formaldehyde or polybenzoxazine resins. Due to their electric conductance, they may find future applications as building materials in communicating fourth generation devices.	Dubey et al. [134]
Cellulose acetate (CA)	Cellulose acetate is a hydrophilic and thermoplastic biodegradable cellulose derivative. It can be conveniently formulated into sheets, nanofibers, etc. CA scaffolds combined with other bioactive molecules, biopolymers, drugs, etc., support endothelial cell migration and adhesion, and do not promote platelet activation. Chemically modified CA mats bolster osteoconduction and osteoinduction and may help bone regeneration.	Laboy-López and Frenández; Shaban et al.; Rubenstein et al. [135,136,137]
Cellulose, bacterial cellulose nanofibrils (CNF)	Cellulose nanofibers (from plant or bacterial sources) are nontoxic, biocompatible, and biodegradable materials that can be produced in large quantities at low cost. Pristine and chemically modified or crosslinked CNFs have applications in controlled drug delivery, antibacterial wound dressing, and skin and bone tissue engineering.	Pandey; Torres et al.; Helenius et al. [138,139,140]
Chitosan	Chitosan is an amino group-containing polysaccharide derived from the natural chitin sources by deacetylation. It contains randomly ordered D-glucosamine and N-acetyl-D-glucosamine units. Chitosan is a highly biocompatible and biodegradable material that can be digested by either lysozyme or chitinase enzymes in the body. It is frequently used for drug delivery, antibacterial wound dressing, tissue engineering, and bone substitution purposes, in combination with other biopolymers like PEGDA, PLA, gelatin, and alginate. The higher degree of deacetylation increases the strength of cell membrane interactions and cellular uptake.	Rodrigues et al.; Venkatesan and Kim; Bojar et al. [141,142,143]
Collagen, Type-I and II	Collagen is a natural fibrous protein with excellent biocompatibility, biodegradability and bioactivity. Type I collagen is the major component of the extracellular matrix and the bones, while Type II collagen can be found in the cartilage tissues. Due to their excellent cellular interactions, both types were applied in bone scaffolds and cartilage repair preparations.	Ferreira et al.; Rezvani Ghomi et al.; Kilmer et al. [2,144,145]
Gelatin	Gelatin is a partly hydrolyzed form of collagen containing interconnecting protein chains. It is isolated from animal skin, bone, or connecting tissues. The amino acid composition and sequence is changing with the origin of the tissue. Gelatin is mostly used with other bioactive polymers, i.e., alginate, chitosan, PLLA, and PCL. In scaffolds, it improves cell adhesion, proliferation, and infiltration.	Su and Wang; Peter et al. [146,147]
Glycosaminoglycan (GAG)	Glycosaminoglycans are long-chained polysaccharides built from repeating disaccharide units. They are present on cell surfaces and in the extracellular matrix. Due to their role in regulating the growth factor signaling, interaction with cytokines, and cell surface receptors, GAGs affect, for instance, the inflammation and cell growth processes. They are used in hydrogels, antibacterial surface layers, and porous scaffolds in tissue engineering.	Köwitsch et al. [148]
Graphene	Graphene nanosheets are made from graphite and consist of only a single layer of carbon atoms. Graphene is biocompatible, although it is not biodegradable. Graphene promotes stem cell growth and proliferation, as well as osteogenic differentiation. High concentrations of pristine graphene may decrease cell viability, but PEGylation may reduce that effect. Due to its electrical conductance, it might find application in the fourth generation of bioactive materials.	Dubey et al. [134]
Graphene oxide (GO)	GO is prepared from graphite or graphene by strong chemical oxidation. Epoxides, hydroxyl, and carboxylic groups are generated on the surface, providing connecting points to anchorage-dependent cells to adhere, spread and function.	Berrio et al.; Dubey et al. [8,134]
Graphene oxide, reduced (rGO)	rGO is made from GO by thermal decomposition or chemical reduction. Epoxide rings are removed, but carboxylic and phenolic groups remain on the perimeter. When combined with collagen type-I, the material becomes mechanically more robust and activates the differentiation of human osteoblast stem cells. Scaffolds made with them could be used in bone substitution.	Bahrami et al.; Norahan et al. [53,149]
Pectin, Methoxyl pectin	Pectin is a highly hydrophilic, biocompatible, and biodegradable natural polysaccharide rich in carboxylic group-containing galacturonic acid. When more than half of the carboxylate groups are in the methyl ester form, the material is called high methoxyl pectin; otherwise, we talk about low methoxyl pectin. High methoxyl pectin can form hydrogels under mildly acidic conditions. Low methoxyl pectins can be crosslinked with calcium ions to make them less polar drug carriers. Pectins are used alone or in combination with other natural polymers in the 3D printing of scaffolds.	Martau et al.; Li et al.; Tortorella et al. [131,150,151]
Poly(lactic-co-glycolic acid) (PLGA)	PLGA is a highly biocompatible and biodegradable material approved by the FDA for drug delivery, gene engineering, and biomedical uses. Pristine polyglycolic acid would hydrolyze readily. Thus, it is blended with PLA or other polymers to improve hydrolytic and degradation properties. PLGA is combined with different bioactive materials (TCP, HA, gelatin, etc.) or bone morphogenetic proteins (BMPs) and is extensively used in artificial bone substitution applications to facilitate cell adhesion and proliferation. PLGA can easily be formulated into various matrices, from solid scaffolds to nanofiber mats.	Makadia and Siegel; Zhao et al.; Elmowafy et al.; Gentile et al.; Jin et al. [152,153,154,155,156]
Poly(lactic acid and poly(L-lactic acid) (PLA and PLLA)	PLA is a highly biocompatible and biodegradable thermoplastic polymeric material approved by the FDA for biomedical, drug delivery, and tissue engineering applications. Due to the less polar nature of PLA, it is frequently used in co-polymers with hydrophilic polyglycolic acid to improve hydrolytic behavior. When pristine PLA is used alone in the body, it often induces foreign body reactions. Electrospun PLA-copolymers and their microspheres and nanoparticles provide bioactive materials for drug delivery, wound healing, or bone substitution. PLA is widely used in 3D printing. In the human body, PLA implants degrade significantly slower than polyglycolic acid.	Makadia and Siegel; Zhao et al.; Elmowafy et al.; Gentile et al.; DaSilva et al.; Tyler et al.; Böstman and Pihlajamaki [152,153,154,155,157,158,159]
Poly(methyl methacrylate) (PMMA)	PMMA is a bioinert polymeric material, the main component of acrylic bone cement. The mechanical properties can be improved by blending, i.e., with polystyrene. PMMA-based bone cement can be injected into the position and cured at room temperature. It can be mixed with antibiotics. PMMA is not biodegradable; it usually works as a spacer in joining implants. Fixation properties can be improved by chemical modification of the PMMA structure and by loading with TCP or other bioactive and degradable materials. PMMA cements are FDA-approved bone graft materials.	Arora; Magnan et al. [160,161]
Poly(ε-caprolactone) (PCL)	PCL is an FDA-approved biocompatible and biodegradable synthetic material for human drug delivery, suture, and adhesion barrier applications. The biodegradation is the slowest among the ester-type bone substitute materials. Thus, PCL is used in long-term implants. Orthopedics frequently combines it with bioactive components like silk fibroin, bioactive glasses, or TCP to improve cell adhesion. It can be formulated by molding, pressing, 3D printing, solution or melt electrospinning.	Janmohammadi and Nourbakhsh; Dwivedi et al. [162,163]
Polybenzoxazine (PBO)	The name polybenzoxazine covers a wide range of polymers in which the benzoxazine/polybenzoxazine moiety is the standard building block. PBO resins are prepared by thermal or catalytic ring opening and polymerization of substituted benzoxazine structures derived from synthetic or natural precursors, i.e., cellulose or chitosan. In thin films, PBOs show good antibacterial and antifungal activity. Carbonization at high temperatures results in carbon foams that offer good biocompatibility.	Ghosh et al.; Periyasamy et al.; Thirukumaran et al.; Lorjai et al. [164,165,166,167]
Poly(ethylene glycol diacrylate) (PEGDA)	Ethylene glycol diacrylate alone or combined with other acrylates can be easily polymerized or photopolymerized to PEGDA and copolymers. Crosslinking may increase the mechanical strength. PEGDA is a hydrophilic and low-immunogenic compound suitable for scaffolds and hydrogels. It is a good drug depot, and the drug release profile can be finely tuned. It can be used in bio-inks for 3D printing to provide biocompatible flow-through devices. It forms hydrogels that are used in cartilage tissue regeneration.	Rekowska et al.; Warr et al.; Qin et al.; Musumeci et al. [168,169,170,171]
Silica	Silica is a biocompatible, biodegradable, and osteoconductive material. Silica enhances the osteogenic differentiation of stem cells and bone regeneration by promoting Type I collagen formation, stabilization, and matrix mineralization. Porous silica can be combined with various polymers, biomaterials, proteins, enzymes, drugs, and hormones. The surface can be covalently functionalized with bioactive agents. Higher concentrations of nano-silica particles may lead to bioaccumulation and cellular damage.	Zhou et al.; Jurkic et al.; Shadjou et al.; Vareda et al. [172,173,174,175]
Silk fibroin	Silk fibroin is a natural protein produced by insects. It is a lightweight but mechanically strong material and can be found, i.e., in spider webs and prepared from the cocoon of the domestic silkworm. Scaffolds made of it are biodegradable, can be functionalized, and support the attachment and growth of cells. In the form of fibers, nanofibers, mats, films, and porous structures, silk fibroin has many applications in cell cultures, tissue engineering, and cartilage tissue regeneration.	Nguyen et al.; Wang et al.; Wang et al.; Farokhi et al. [176,177,178,179]
Starch	Starch is a natural polysaccharide consisting of d-glucose units. It is produced mainly from potatoes, manioc, or seeds like rice, wheat, and corn. Starch is an edible, biocompatible, and readily biodegradable material. It supports cell growth on the surface. It can be formulated in different shapes and porosities with biodegradable polymeric materials. By 3D prototyping, custom-shaped bioactive scaffolds are created.	Martins et al.; Salgado et al. [180,181]
Strontium ranelate (SR)	SR is a medical drug to treat osteoporosis in men and women, regardless of age. It is capable of reducing the risk of fracture. Strontium ranelate promotes the osteoblastic differentiation of stem cells, inhibits osteoclasts, and improves the structure of bones.	Pilmane et al.; Kaufman et al.; Cianferotti et al. [182,183,184]
Tricalcium phosphate (βTCP, TCP)	Beta tricalcium phosphate is the “gold standard” of bone grafts approved by the FDA. It is osteoinductive, biodegradable, and one of the most extensively used bone substitute materials in clinical practice. The physical appearance of TCP covers a wide range, from low-strength porous bodies to hard grafts. TCP shows no adverse effects and maintains normal calcium and phosphate ions level in the blood. The apparent in vivo behavior is affected to some extent by the purity and the way TCP was produced. TCP is insoluble under physiological conditions at pH 7.4 and is dissolved and resorbed by cell-mediated processes. The resorption time is in the 6–24 month range.	Lu et al.; Bohner et al.; Tanaka et al.; Gilmann and Jayasuriya [185,186,187,188]
Xanthan gum	Xanthan gum is a biodegradable branched polysaccharide produced in large quantities by industrial fermentation with the bacteria Xanthomonas campestris. The backbone is cellobiose, and the branches contain D-mannoses and D-glucuronic acid. The structure of the chain in solutions can be tuned from coiled to helical by increasing the temperature and the ionic strength. High-molecular-weight xanthan gums, frequently in combination with other biopolymers, have found application in the biomedical field, from drug delivery to bone substitute scaffolds.	Petri [189]

## Data Availability

No new data were created or analyzed in this study. Data sharing is not applicable to this article.
